# Investigation of Various Toxigenic Genes and Antibiotic and Disinfectant Resistance Profiles of *Staphylococcus aureus* Originating from Raw Milk

**DOI:** 10.3390/foods13213448

**Published:** 2024-10-29

**Authors:** Gulay Merve Bayrakal, Ali Aydin

**Affiliations:** Department of Food Hygiene and Technology, Faculty of Veterinary Medicine, İstanbul University-Cerrahpaşa, İstanbul 34320, Türkiye; aliaydin@istanbul.edu.tr

**Keywords:** antibiotic resistance, disinfectant resistance, raw milk, *Staphylococcus aureus*, toxigenic genes

## Abstract

This study investigated the toxigenic genes and antimicrobial resistance profiles of *Staphylococcus aureus* strains isolated from 260 raw milk samples collected from dairy farms in Türkiye. The results indicated that 60.7% of staphylococcal enterotoxin genes (*sea*, *seb*, *sed*, *seg*, *sei*, *sej*, *sek*, *seq*, *sem*, *seo*, and *seu*) and 21.4% of the *tst* and *eta* genes were positive, with most enterotoxin-positive samples carrying more than one gene. The *sec*, *see*, *seh*, *sel*, *sen*, *sep*, and *etb* genes were not identified in any samples. The prevalence of antibiotic resistance genes (*mecA*, *blaR*, *blaI*, *blaZ*, *vanA*, *ermT*, *tetK*, *aac*/*aph*, *ant*, *dfrA*, *tcaR*, *IS256*, and *IS257*) was high at 89.2%, with *bla* being the most frequently detected gene (75%). The *mecA* gene was present in 14.2% of samples, while *tcaR* was detected in 78.5%. Nevertheless, the *mecC* was not identified. Disinfectant resistance genes (*qac*A/B, *qacC*, *qacJ*, *smr*) were detected in 21.4% of the samples. The results of the disk diffusion test showed that 64.2% of strains were resistant to penicillin G and ampicillin, with additional resistance found for cefoxitin, teicoplanin, levofloxacin, norfloxacin, and other antibiotics. These findings highlight a significant public health and food safety risk associated with raw milk due to the presence of *S. aureus* strains with toxigenic genes and high antimicrobial resistance.

## 1. Introduction

Milk is a nutritionally complete food, providing all the nutrients required for human growth and healthy development. Nevertheless, raw milk is also an optimal source of nutrition for the cultivation of pathogens and spoilage organisms [1]. As the demand for raw milk, perceived to be more nutritionally beneficial by the public, continues to rise, a corresponding increase in health concerns has been observed. *Staphylococcus aureus* (*S. aureus*) is one of the most significant pathogenic microorganisms found in milk and is responsible for several serious human diseases [2,3]. In fact, an analysis of studies conducted between 1992 and 2021 showed that the prevalence of *S. aureus* was higher in raw milk than in pasteurized milk and dairy products [4]. Due to virulence factors such as antimicrobial resistance, *S. aureus* is an important pathogen for dairy farms.

*S. aureus* is dairy cattle’s most frequently identified causative agent of clinical and subclinical mastitis [5,6]. It is worth noteworthy that, in addition to mastitis in animals, *S. aureus*, which can be transmitted from farm personnel to milk, has been identified as a causative agent of human food poisoning [7]. In contrast to other toxins secreted by *S. aureus*, a relatively small quantity is sufficient for enterotoxins to exert their toxic effects. Staphylococcal food poisoning is a significant cause of foodborne illness globally, attributed to enterotoxins with enhanced tolerance to environmental agents [8,9]. In addition, the World Health Organization (WHO) reported that *S. aureus* poisoning was 77.3 per 100,000 population for 61 European and other low-mortality countries [10].

*S. aureus* possesses a variety of virulence factors that contribute to its pathogenicity [3]. The known secretory virulence factors include toxins (staphylococcal enterotoxins, toxic shock syndrome toxin-1 (*tst*), hemolysins, and exfoliative toxins A and B (*eta* and *etb*) [3,11]. Due to its virulence properties, *S. aureus* can overcome the host defense system, thereby facilitating the occurrence of disease, and prolonging the course of treatment. 

Food represents a significant vector for the transmission of antimicrobial resistance [7]. The term “antimicrobial resistance” encompasses both disinfectant and antibiotic resistance. The emergence of penicillin and methicillin resistance is frequently observed because of excessive antibiotic treatment [12]. Methicillin-resistant *S. aureus* (MRSA) represents a significant global public health threat worldwide, particularly in humans and animals, and is a common cause of severe infection [13]. Methicillin resistance has increased alarmingly over the years, and the median rate of MRSA-causing bloodstream infections is 35% [12]. In addition, the prevalence of *S. aureus* strains in food has been reported to be 72.94% in our country [14]. The primary cause of hospital-acquired infections, MRSA, exhibits resistance to numerous known antibiotics, rendering the fight against the disease even more challenging. As a result of antibiotic resistance, antibiotics become ineffective, and infections become difficult or impossible to treat. This increases the risk of disease spread, serious illness, disability, and death [12,15].

Quaternary ammonium compounds (QACs) are commonly used disinfectants in the dairy industry, employed for disinfecting milking equipment and udder disinfection, particularly for mastitis prevention [16]. In the absence of the requisite conditions, including the correct selection of disinfectant, appropriate dosage, and pre-cleaning, the efficacy of disinfectants is diminished, leading to the emergence of resistant strains [17]. This situation represents a significant public health concern that requires urgent attention. 

This study investigated the virulence characteristics and antimicrobial resistance profile of *S. aureus* strains isolated from raw milk. The antimicrobial resistance profiles of *S. aureus* strains isolated from raw milk were investigated by analyzing the presence of antibiotic and disinfectant resistance genes. Moreover, agar diffusion assays were performed to evaluate the antibiotic susceptibility of the samples against nine distinct antibiotic groups. To identify the virulence properties, an investigation was conducted into the presence of SEs genes responsible for enterotoxin production, exfoliative toxin genes (*eta* and *etb*), and the toxic shock syndrome toxin-1 gene (*tst*). Furthermore, the milk to be analyzed in the study was collected from the Thrace region, which represents a border area with Europe. The objective was to examine a region that is significant regarding disease control.

## 2. Materials and Methods

### 2.1. Sampling, Isolation, and Identification of S. aureus 

A total of 260 raw milk samples from 7 different farms (a, b, c, d, e, f, g) from 3 different regions [Ipsala region (farms a, b, c), Kesan region (farm e), Luleburgaz region (farms d, f, g)] in the Thrace area of Türkiye were randomly selected. Each sample was collected from one dairy cow, and the raw milk samples were collected in sterile falcons (50 mL, Isolab, 078.02.004, İstanbul, Türkiye) under aseptic conditions and transferred to the laboratory at a cold temperature (+4 °C). 

The milk samples plated on Baird Parker Agar (BPA, Oxoid, CM275, Basingstoke, UK) and *S. aureus* were isolated. The isolation of *S. aureus* from raw milk samples was conducted using EN ISO 6881-1 standardized procedures documented by the International Organization for Standardization [18]. The strains were confirmed by biochemical tests, including gram staining, catalase testing, latex agglutination testing, growth on mannitol fermentation (using Mannitol Salt Agar, Oxoid CM0085B, Oxoid Ltd., Hampshire, UK), and DNAse activity testing (using DNAse agar, Oxoid CM0321, Oxoid Ltd., Hampshire, UK). 

*S. aureus* strains isolated by conventional methods were then confirmed by PCR after DNA extraction [7]. To verify the identity of the *S. aureus* strains, an analysis was conducted to determine the presence of the thermonuclease gene (*nuc*), the coagulase gene (*coa*), and the production of the surface protein A gene (*spa*) by PCR [7,19,20].

### 2.2. Detection of Toxigenic Genes in *S. aureus* Isolates 

The presence of staphylococcal enterotoxin genes *sea*, *seb*, *sec*, *sed*, *see* [21], *seg*, *seh*, *sei*, *sej*, *sek*, *sel*, *sep*, *seq* [22], *sem*, *sen*, *seo* [23], and *seu* [24] were detected using multiplex and monoplex PCR. Moreover, the presence of the exfoliative toxins’ genes (*eta*, *etb*) [21], and the toxic shock syndrome toxin-1 gene (*tst*) [25] were investigated.

### 2.3. Determination of Antimicrobial Susceptibility of *S. aureus* Isolates

The agar disk diffusion method determined the isolates’ antimicrobial susceptibility by the guidelines set forth by the European Committee on Antimicrobial Susceptibility Testing (EUCAST) [26]. The following antibiotic disks were used: penicillin G (Oxoid-CT0043, 10 U, Oxoid Ltd., Hampshire, UK), ampicillin (Oxoid-CT0003, 10 μg), gentamicin (Oxoid-CT0024, 10 μg), tobramycin (Oxoid-CT0056, 10 µg), teicoplanin (Oxoid-CT0647, 30 μg), ceftaroline (Oxoid-CT1942, 5 μg), cefoxitin (Oxoid-CT0119, 30 μg), tetracycline (Oxoid-CT0054, 30 µg), erythromycin (Oxoid-CT0020, 15 μg), levofloxacin (Oxoid-CT1587, 5 µg), ofloxacin (Oxoid-CT0446 5 μg), norfloxacin (Oxoid-CT0434, 10 µg), fusidic acid (Oxoid-CT0023, 10 μg), trimethoprim-sulfamethoxazole (Oxoid-CT0052, 1.25 μg/23.75 μg), and linezolid (Oxoid-CT1649, 10 μg). The antibiotics were selected from nine preferred antibiotic groups, namely penicillins, aminoglycosides, glycopeptides and lipoglycopeptides, cephalosporins, tetracyclines, macrolides, lincosamides and streptogramins, fluoroquinolones, miscellaneous agents, and oxazolidinones. Antimicrobial disks were placed on Mueller–Hinton agar (MHA, CM 337 Oxoid) containing *S. aureus*. Following the incubation period, the diameter of the inhibition zone was measured and evaluated. MDR isolates were defined as those exhibiting resistance to at least three distinct antimicrobial classes.

### 2.4. Detection of Antimicrobial Resistance Genes in *S. aureus* Isolates

Multiplex and monoplex PCR was used to detect the presence of antibiotic resistance genes, including methicillin resistance genes (*mecA* [27] and mecC [28]), penicillin resistance genes (*blaR*, *blaI*, *blaZ* [*blaZ*F-R]) [29], and (*blaZ*1-2) [30], aminoglycoside resistance genes (*aac*/*aph*, *aph*, *ant*) [31], teicoplanin-associated locus (*tcaR*) [32], vancomycin resistance gene (*vanA*) [33], trimethoprim resistance gene (*dfrA*) [29], erythromycin resistance gene (*ermT*) [34], tetracycline resistance gene (*tetK*) [29], and insertion sequence (IS) elements (*IS256*, *IS257*) [29]. Isolates were classified as multidrug-resistant when they exhibited an inability to respond to at least three distinct drug classes. To detect disinfectant resistance, which is the other responsible factor for the formation of antimicrobial resistance, the presence of the QAC resistance gene *qacAB* and *smr* [35], *qacC* [29], and *qacJ* [36] was determined by monoplex PCR.

The PCR products were analyzed by horizontal electrophoresis in a system containing 1xTris-acetate-EDTA (TAE) buffer, 1.5% (*w*/*v*) agarose gel, and 5% (*v*/*v*) fluorescent DNA dye (SafeView Classic, Applied Biological Materials Inc., Richmond, BC, Canada). The gels were imaged using the Infinity Gel Imaging System (Vilber Lourmat, Marne-la-Vallée, France). All the PCR experiments were performed twice for each strain.

## 3. Results

### 3.1. Toxigenic Genes (Virulence Genes) 

In this study, 260 raw milk samples were collected, and 28 strains (10.7%) of *S. aureus* were confirmed by PCR following a microbiological analysis. Table 1, Table 2 and Table 3 present the investigation of virulence and antimicrobial resistance genes in these strains. 

The research findings indicated that at least one gene encoding for enterotoxin was present in 60.7% of the *S. aureus* samples obtained from raw milk. Fourteen of the 17 enterotoxin-positive samples demonstrated the presence of two or more distinct enterotoxin genes. The most prevalent gene observed in the samples was the *seo* gene (35.7%), and the gene observed in three samples carrying a single gene was also the *seo* gene. The percentages of the *sea*, *seb*, *sed*, *seg*, *sei*, *sej*, *sek*, *sem*, *seo*, *seu* and *seq* enterotoxin genes that were isolated from the 28 samples were found to be 7.1%, 17.8%, 17.8%, 25%, 28.5%, 3.5%, 7.1%, 25%, 35.7%, 32.1% and 17.8%, respectively. The *sec*, *see*, *seh*, *sel*, *sen*, and *sep* genes were not identified in any samples. When the virulence characteristics of *S. aureus* isolates were analyzed, it was found that they did not differ significantly according to region or farm. The *seo* gene was not observed in farms c (Ipsala region), and d (Luleburgaz region), and the *tst* gene was not observed in farms d and f (Luleburgaz region). While only 1 sample from farm a (Ipsala region) carried the *seo* gene, 80% of the samples collected from farm e (Kesan region) carried at least five enterotoxin genes. 

Regarding the virulence genes examined, the *tst* gene was identified in 21.4% of the samples. Among the exfoliative toxin genes, the *eta* gene was identified in a single sample (3.5%), while the *etb* gene was not detected. 

### 3.2. Antimicrobial Susceptibility 

The antimicrobial susceptibility of the isolates was determined by the agar disk diffusion method. The results of the disk diffusion test indicated that the highest resistance was observed against penicillin G and ampicillin (64.2%), followed by cefoxitin (28.5%), teicoplanin (21.4%), levofloxacin (21.4%), and norfloxacin (17.8%). The prevalence of resistance to ofloxacin and fucidic acid was 14.2%, while the prevalence of resistance to tobramycin, tetracycline, and erythromycin was 7.1%. The lowest resistance was observed in the gentamicin treatment group. All isolates demonstrated susceptibility to ceftaroline, trimethoprim-sulfamethoxazole, and linezolid.

### 3.3. Antimicrobial Resistance Genes 

This study demonstrated a high prevalence (89.2%) of antimicrobial resistance genes in *S. aureus* strains isolated from raw milk. The analysis of antibiotic resistance genes revealed the presence of the *mecA* gene, responsible for resistance to the methicillin antibiotic, at a rate of 14.2%. In contrast, the *mecC* gene was not detected in any examined strains. The most frequently identified antibiotic resistance gene was the *bla* gene, which is responsible for resistance to beta-lactam antibiotics. This gene was identified in 75% of the samples analyzed. In this study, the *aac*/*aph* and *ant* genes responsible for the development of resistance to the aminoglycoside antibiotic group were identified to be present in 3.5% and 7.1% of the strains, respectively. The *aph* gene was not detected in any of the strains. The tcaR gene, responsible for resistance to the teicoplanin antibiotic, was identified in 78.5% of the isolates. The vanA gene, responsible for vancomycin resistance, was analyzed at a rate of 17.8%. In contrast, the dfrA gene, responsible for resistance to trimethoprim antibiotics, was studied at a rate of 7.1%. The *ermT* gene, responsible for resistance to the erythromycin antibiotic from the lincosamide group, and the *tetK* gene, responsible for tetracycline resistance, were identified in a single sample each. The insertion sequence (IS) elements IS256 were identified in 53.5% of the cases, while IS257 was analyzed in 3.5%.

The QAC genes responsible for QAC disinfectant resistance were identified in 21.4% of the samples. The prevalence of disinfectant resistance genes *qacAB*, *qacC*, *qacJ*, and *smr* was determined to be 7.1%, 7.1%, 17.8%, and 21.4%, respectively. It was observed that three (75%) of the samples carrying *mecA* resistance gene also carried disinfectant resistance gene. All samples carrying the disinfectant resistance gene carried the *bla* gene and 83.3% of them carried the *tcaR* gene. In addition, it was observed that all samples carrying disinfectant resistance genes carried at least three antibiotic resistance genes and were MDR isolates. 

Regarding the differences between the farms, no disinfectant resistance gene was detected in the samples from farms a (Ipsala region), e (Kesan region), and g (Luleburgaz region). No antibiotic resistance gene was detected in the sample from farm g, and this sample was only resistant to the antibiotic ampicillin. None of the samples from farm f (Luleburgaz region) carried the *bla* gene, whereas almost all the samples from the other farms carried the *bla* gene. In addition, 80% of the samples from farm f were resistant to the antibiotic teicoplanin. 

## 4. Discussions

### 4.1. Toxigenic Genes 

Epidemiological studies have demonstrated that *S. aureus* strain agents in milk produce a group of virulence factors and have indicated a correlation between the severity of infection and the virulence factors produced by *S. aureus* [3]. The most significant virulence factor of *S. aureus* is the production of enterotoxin. SEs are regarded as an important public health concern [37]. Several studies have investigated the occurrence of enterotoxin genes in strains of *S. aureus* isolated from food sources, reporting a range of prevalence rates for different genes. In their study, Zhang et al. reported the detection of the *sea* (17.45%), *seb* (16.44%), *sec* (7.38%), and *sed* (1.68%) enterotoxin genes, while the *see* gene was not detected [3]. In their 2020 study, Titouche et al. reported that 62.5% of the samples tested positive for at least one gene encoding SEs. The most prevalent genes were *sei* and *seg* (47.69%), followed by *seb* (23.08%) [38]. Notably, none of the isolates carried the *sed* and *see* genes. In another study, the *seg*, *sei*, and *sec* genes were identified as positive in 35% of raw milk samples, while the *sea* and *sei* genes were found to be harmful [39]. In a study conducted by Khemiri et al., at least one enterotoxin gene was identified in 87.5% of *S. aureus* strains isolated from raw milk samples [40]. Of these, the *sed* gene was the most frequently analyzed. Consistently with previous studies [3,38,39], our analysis revealed the presence of at least one enterotoxin gene in the strains under investigation, while the *sec* and *see* genes were not identified. In contrast with this study’s findings, Fursova et al. (2018) examined the *sea* gene in 53.30% and the *sec* gene in 50% of *S. aureus* strains isolated from food sources [41]. Furthermore, this study identified the presence of the *seb*, *sed* (17.8%), and *sei* (28.5%) genes, which were not detected in the studies conducted by Adame-Gómez et al. and Pereira et al. (2009) [39,42]. One of the primary causes for the disparate outcomes observed in the study was attributed to the distinct characteristics exhibited by the strains in varying geographical regions. As observed in our study, the *see* gene was not detected, while the *sed* gene was among the most prevalent in a similar study conducted in the Thrace region by Papadopoulos et al.. The findings of our study are consistent with those of a similar investigation conducted in our neighboring country, the Thrace region. The *see* gene was not detected in the study, while the *sed* gene was identified with high frequency [43].

In addition to food poisoning, *S. aureus* is a causative agent of a range of acute and chronic diseases, including septicemia, pneumonia, endocarditis, and respiratory tract illnesses, as well as autoimmune diseases. These diseases have a high morbidity rate in a variety of hosts. One of these diseases is toxic shock syndrome [44]. In their study, Zhang et al. [3] identified the presence of the *tst* gene at 23.50%, while Dan et al. [45] reported a rate of 26.5%. Conversely, the *tst* gene determined in 21.4% of cases in this study. In contrast with the findings of our study, the *tst* gene was not identified in other studies conducted on milk [3,42,46].

Exfoliative toxins (*eta*, *etb*) are virulence factors secreted by staphylococci. These toxins are responsible for the degradation of keratinocytes in both human and animal skin, which can result in the development of skin infections [47]. In this study, the *eta* gene was identified as positive, whereas the *etb* gene was identified as negative. In other studies, Tegegne et al. (2021) analyzed the *eta* gene in 22.05% of *S. aureus* strains isolated from milk [2]. Similarly, Kot et al. (2016) analyzed the *eta* gene in 5.6% of *S. aureus* strains isolated from milk [48]. The studies conducted by Dan et al., Gharsa et al., and Chenouf did not identify the presence of the *eta* and *etb* genes in any of the samples [45,49,50]. The findings demonstrate that *S. aureus* strains may encode a range of virulence factors and that the distribution of these factors varies between different genotypes. These results indicate that *S. aureus* virulence is not exclusive to the strain level but is also influenced by factors such as the origin and genetic background of the strain [45]. 

### 4.2. Antimicrobial Susceptibility 

Upon examination of the antibiotic resistance profiles, it was observed that the results of the disk diffusion and resistance genes assays were consistent for the beta-lactam group. In the course of our investigation, it was found that 75% of the isolates encoding the *bla* gene and 64.2% of the isolates were resistant to penicillin G. The *aac/aph* and *ant* genes responsible for resistance to the aminoglycoside group were detected in 3.5% and 7.1% of isolates, while resistance to gentamicin and tobramycin was 3.5% and 7.1%, respectively. The findings of our study are from other researchers, indicating that the highest resistance was observed in the penicillin group [39,45,51]. Dan et al. observed, as our study revealed, that the isolates did not resist linezolid [45]. However, in contrast to the findings of this study, the isolates exhibited high resistance to teicoplanin. As previously observed by Pereira et al. (2009), a minor percentage of isolates showed resistance to gentamicin, erythromycin, and tetracycline [39]. Our study reports a lower prevalence of norfloxacin-resistant isolates than that Kotb and Gafer observed [52]. 

The data presented here are similar to those reported by Dan et al., in that all MRSA isolates demonstrated resistance to penicillin G and ampicillin. Of the analyzed samples, 78.5% showed resistance to more than one antibiotic [45]. The findings of our study indicate that some isolates encoding resistance genes (*tcaR* and *dfrA*) did not develop resistance to the antibiotic. These results suggest that, despite the presence of antimicrobial resistance genes, their expression may be insufficient to confer a resistant phenotype. Nevertheless, these strains have the potential to transform into resistant strains under certain conditions and may therefore have epidemiological importance in the transmission of antibiotic resistance [45].

### 4.3. Antimicrobial Resistance Genes 

The increasing prevalence of antimicrobial resistance in *S. aureus* represents a significant global public health concern [4]. Due to the excessive and inappropriate usage of antimicrobials, microorganisms develop resistance to these substances, transferring this resistance to other microorganisms through the transfer of genes [53]. This situation causesconcern about both food safety and public health. The range of treatment options for life-threatening infections caused by MDR strains of *S. aureus* is limited. Considering these considerations, the WHO classified *S. aureus* as the most significant bacterial pathogen exhibiting antibiotic resistance in 2017 [54].

Methicillin-resistant *S. aureus* is one of the most significant antibiotic-resistant bacteria. Isolates of MRSA are frequently multidrug-resistant, which results in increased costs, longer treatment durations, and higher rates of hospitalization and comorbidity. The *mecA* gene, which encodes the production of penicillin-binding proteins, is responsible for methicillin resistance in the MRSA chromosome It is, therefore, considered the most reliable method for detecting methicillin resistance [4,55]. This study revealed the presence of the *mecA* gene in 14.2% of the samples. A review of the literature on the detection of the *meC* gene in milk showed that the findings of Dan et al. were consistent with those of this study [45]. The results of the *mecA* analysis in this study were found to be lower than those reported by Ganai et al. [56], Riva et al. [57], and our neighboring country [43], but higher than those observed by Mahanti et al. [58], Chenouf et al. [50], and Pereira et al. [39]. In studies conducted in Türkiye, the prevalence of strains carrying the *mecA* gene was found to be 6.3% [59] and 1.70% [60], which is lower than the data obtained in this study. In numerous additional studies conducted in Türkiye, the *mecA* gene was not detected in *S. aureus* strains isolated from food sources [51,61,62]. It is also noteworthy that, as with this study, the *mecC* gene was not detected in studies conducted in Brazil [63] and Greece [43]. 

The primary mechanism of resistance to penicillin and penicillin derivatives is the *blaZ* gene, which encodes the production of beta-lactamases that hydrolytically break down beta-lactams [4,55]. Elevated levels of the *blaZ* gene have been reported in milk (74.07%) [63], (94.6%) [64], (95.7%) [65]. In this study, the *blaI* and *blaR* genes, which are responsible for beta-lactamase production, were also analyzed. A literature review revealed no previous study of these genes in raw milk in Türkiye. The *blaI* and *blaR* genes regulate the expression of the *blaZ* gene [66]. In a study by Kreausukon et al., the presence of the blaI, blaR, and blaZ genes was identified in all MRSA strains [67]. Additionally, the study demonstrated elevated levels of the blaI and blaR genes. Furthermore, the investigation revealed that the blaI and blaZ genes were present in all isolates that also carried the blaR gene.

The bifunctional enzyme AAC/APH encoded by the aac/aph gene, the APH enzyme encoded by the aph gene, and the ANT enzyme encoded by the ant gene are responsible for resistance to aminoglycosides [68]. This study identified the presence of the aac/aph and ant genes in various strains, while the aph gene was not detected in any strains. In other studies, the presence of these genes in milk was confirmed [68,69]. The Teicoplanin-associated locus (tcaR) gene, which is associated with teicoplanin resistance, was analyzed for the first time in Türkiye and the results demonstrated that it was present in 78.5% of the samples examined. In contrast, the tcaR gene was identified in clinical isolates in a study conducted in India [70]. 

Vancomycin, a glycopeptide antibiotic, has been employed in treating MRSA for several decades. This has resulted in the emergence of vancomycin-resistant S. aureus strains [71]. In this study, the prevalence of the vanA resistance gene was determined to be 17.8%, which is consistent with the findings of Kou et al. [72]. The dihydrofolate reductase protein, which confers resistance to trimethoprim (dfrA), was identified at a prevalence of 7.1% in our study. Nevertheless, the dfrA gene was not detected in raw milk in Egypt [73] and Mozambique [74], nor in chicken, fish, and red meat in Türkiye [75]. 

The resistance to lincosamides, including erythromycin and clindamycin, is a consequence of the methylation of the receptor-binding site on the ribosome. Methylation is a process mediated by an enzyme called methylase and encoded by erm genes. During our investigation, the ermT gene was isolated from a single isolate. Nevertheless, other studies have indicated that the ermT gene was not identified [50]. The efflux pump system encoded by the tetK gene and carried by plasmids represents one of the resistance pathways to tetracyclines, a broad-spectrum antibiotic [71]. The prevalence of the tetK gene, which was identified in a single isolate in our study, was found to vary significantly in other studies (30%) [50], (3.2%) [45], (48.66%) [4].

The insertion sequence (IS) elements IS256 and IS257, which are associated with multiple antibiotic resistance, were identified as present in this study. As observed in our data, studies by Zhang et al. and Miao et al. similarly identified the presence of IS257 and IS256, respectively [76,77]. Araújo et al. did not identify the presence of IS256 and IS257 in their samples [78]. This study revealed that 64.2% of *S. aureus* strains demonstrated multidrug resistance (MDR), predominantly to beta-lactams, methicillin, and teicoplanin [Table 3]. The findings indicated a high prevalence of MDR *S. aureus* in raw milk, representing a significant public health concern. Absent the implementation of necessary preventive measures, the probability of encountering far more significant difficulties in the future is considerable. To prevent the development and transmission of antibiotic-resistant strains, it is recommended that different groups of antibiotics be preferred to this resistance group of antibiotics, mainly when antibiotics are used in dairy farms, and that the misuse and overuse of antimicrobials be avoided.

A further component of antimicrobial resistance is disinfectant resistance. To investigate the potential for disinfectant resistance, this study examined the presence of the efflux pump gene (*qac* and *smr*), which is responsible for resistance to QACs. The *qac* and *smr* genes resulted in 21.4% positive results. The findings of Kroning et al. [17], Kotb and Gafer [52] and Ergun et al. [79] indicate a lower prevalence of the *qac* gene than that observed in our data. Furthermore, the *qacJ* gene, identified as 17.8% positive in our study, was found to be negative by Kroning et al. [17]. An analysis of the study results showed that all samples with disinfectant resistance genes also carried at least three antibiotic resistance genes and were MDR isolates.

Three of the four MRSA strains were found to carry a disinfectant resistance gene. This was regarded as an additional risk associated with MRSA strains. Türkiye ranks first in the world among European and OECD countries regarding antibiotic resistance. The results show that the problem of antibiotic resistance continues. The reasons for the high level of antibiotic resistance in our country include not paying attention to the duration of antibiotic use (shorter or longer than necessary), the preference for broad-spectrum antibiotics, the easy access to antibiotics, and the use of antibiotics without veterinary control. 

Furthermore, previous research has indicated a genetic correlation between disinfectant and antibiotic resistance genes [80,81]. The co-detection of genes responsible for both disinfectant and antibiotic resistance in strains suggests the presence of antimicrobial-resistant strains. Despite the disparate mechanisms of action exhibited by the two groups of genes, genetic studies have revealed striking similarities in their genetic systems and the locations of their genes on the same mobile genetic elements [80]. Moreover, research has indicated that the probability of the pathogen developing antibiotic resistance increases with prolonged exposure to the disinfectant [82]. As using QAC group disinfectants for a long time and at low/high concentrations in the same farm leads to the development of resistance, using different types of disinfectants at recommended doses during specific periods may be effective in preventing resistance. 

## 5. Conclusions

This study represents the first comprehensive investigation to combine virulence factors such as antibiotic and disinfectant resistance levels with enterotoxigenic genes of raw milk in Türkiye. The high prevalence of virulence and antimicrobial genes in *S. aureus* in our study indicates a potential risk associated with raw milk. The fact that *S. aureus*, a vital food pathogen, has high virulence properties in raw milk and is a carrier of enterotoxin genes, among the most important causes of food poisoning, shows that it is a serious food safety and public health problem. Necessary measures must be taken to eliminate this situation, representing a significant risk to food safety and public health. Dairy products should be routinely tested for resistant strains of *S. aureus*. To prevent the development of enterotoxins and disinfectant resistance, it is essential to implement hygiene and sanitation procedures and ensure the correct selection of disinfectants. Risk assessment, Hazard Analysis and Critical Control Points (HACCP), and Good Hygienic Practice (GHP) approaches must be followed.

Global action should be taken against MDR bacteria and the development of antimicrobial resistance. To prevent the growth of MDR bacteria, which represents a significant potential health threat in the future, it is essential to focus on the selection of antibiotics and the utilization of novel, advanced-generation antibiotics. After correctly diagnosing the disease in the animals on the farm, the right antibiotic for that disease should be selected. The use of antibiotics on dairy farms should be more strictly controlled. Suppose the necessary precautions are not taken regarding the development of antimicrobial resistance, which is very important for the future of dairy farming, food safety, and public health. In that case, it is predicted that we will face much more severe problems. Further studies are needed to determine the risks associated with food and the precautions to be taken before these risks occur. Further studies should be conducted to investigate the relationship between disinfectants and antimicrobial resistance and alternative interventions (disinfection methods, etc.) to reduce the prevalence of *S. aureus* in raw milk.

## Figures and Tables

**Table 1:** The results of the virulence gene profile analysis of *S. aureus* strains.

Strain Number (*n* = 28)	Farms	Virulence Gene	
		Enterotoxin-Producing Gene	Toxic Shock Syndrome Toxin-1 Gene and Exfoliative Toxin Genes
2	a		*tst*
4	a		
10	a		
18	a	*seo*	*tst*
22	b		
23	b	*seo*, *seu*, *seq*	
24	b	*sed*, *sei*, *sej*, *sek*, *seo*, *seq*	
25	b	*seu*, *seq*	
26	b	*sei*, *seo*	*tst*
29	b	*sei*, *seu*	
43	b		
44	b	*seo*	
46	c	*sea*, *sed*, *sek*, *sem*, *seq*	*tst*
47	c	*sea*, *sed*, *seq*	
97	d		
100	d		
101	d	*seb*, *sed*, *seg*, *sei*, *sem*, *seu*	
102	d		
112	e	*seb*, *seg*, *sei*, *sem*, *seo*, *seu*	
114	e	*seb*, *seg*, *sei*, *sem*, *seu*,	
123	e	*seb*, *seg*, *sei*, *sem*, *seo*, *seu*	
124	e	*seb*, *seg*, *sei*, *sem*, *seu*	
131	e		*tst*
167	f	*seo*	
168	f		
190	f		
194	f	*seg*, *seo*	*tst*, *eta*
218	g	*sed*, *seg*, *sem*, *seo*, *seu*	
Total Strain Number (%)		17/28 (60.7%)	6/28 (21.4%)

**Table 2:** Distribution of antibiotic-resistant strains of *S. aureus*.

Antibiotic Groups	Name of Antibiotics	Distribution of *S. aureus* Strains According to EUCAST	
		R	S
		(%)	(%)
**Penicillins**	Penicillin G 10 µg	18	10
		(64.2%)	(35.7%)
	Ampicillin 10 µg	18	10
		(64.2%)	(35.7%)
**Aminoglycoside**	Gentamicin 10 µg	1	27
		(3.5%)	(96.4%)
	Tobramycin 10 µg	2	26
		(7.1%)	(92.8%)
**Glycopeptides and Lipoglycopeptides**	Teicoplanin 30 µg	6	22
		(21.4%)	(78.5%)
**Cephalosporins**	Ceftaroline 5 µg	0	28
		(0)	(100%)
	Cefoxitin 30 µg	8	20
		(28.5%)	(71.4%)
**Tetracycline**	Tetracycline 30 µg	2	26
		(7.1%)	(92.8%)
**Macrolides, lincosamides, and streptogramins**	Erythromycin 15 µg	2	26
		(7.1%)	(92.8%)
**Fluoroquinolones**	Levofloxacin 5 µg	6	22
		(21.4%)	(78.5%)
	Ofloxacin 5 µg	4	24
		(14.2%)	(85.7%)
	Norfloxacin 10 µg	5	23
		(17.8%)	(82.1%)
**Miscellaneous Agents**	Fusidic Acid 10 µg	4	24
		(14.2%)	(85.7%)
	Trimethoprim-Sulfamethoxazole (1.25 μg/23.75 μg)	0	28
		(0)	(100%)
**Oxazolidinones**	Linezolid 10 µg	0	28
		(0)	(100%)

**Table 3:** The results of the antimicrobial resistance gene and antimicrobial susceptibility test.

Strain Number (*n* = 28)	Farm	Antimicrobial Resistance Gene		Antimicrobial Susceptibility Test
		Disinfectant Resistance Gene	Antibiotic Resistance Gene	
2	a		*BlaI*, *BlaZ*1-2, *tcaR*, *IS256*	Penicillin G, Ampicillin
4	a		*BlaI*, *BlaZ*1-2, *tcaR*, *IS256*	Penicillin G, Ampicillin
10	a		*BlaR*, *BlaI*, *BlaZ*1-2, *BlaZ*F-R, *tcaR*, *IS256*	Ampicillin, Cefoxitin, Fusidic Acid
18	a			
22	b		*BlaI*, *BlaZ*1-2, *BlaZ*F-R, *tcaR*, *vanA*	Penicillin G, Ampicillin, Levofloxacin, Norfloxacin
23	b	*qacC*, *qac*J, *smr*	*BlaR*, *BlaI*, *BlaZ*1-2, *BlaZ*F-R, *tcaR*, *vanA*, *dfrA*, *ermT*, *IS256*	Penicillin G, Ampicillin, Cefoxitin, Erythromycin, Levofloxacin
24	b	*qacJ*, *smr*	*BlaR*, *BlaI*, *BlaZ*1-2, *BlaZ*F-R, *tcaR*, *IS256*	Penicillin G, Levofloxacin, Ofloxacin, Norfloxacin
25	b		*BlaI*, *BlaZ*1-2, *BlaZ*F-R, *tcaR*, *dfrA*	Penicillin G, Levofloxacin, Ofloxacin, Norfloxacin
26	b		*BlaI*, *BlaZ*1-2, *BlaZ*F-R, *ant*, *tcaR*, *IS256*	Cefoxitin, Fusidic Acid
29	b		*BlaR*, *BlaI*, *BlaZ*1-2, *BlaZ*F-R, *tcaR*, *IS256*	Penicillin G, Ampicillin, Levofloxacin, Norfloxacin
43	b		*BlaI*, *BlaZ*1-2, *BlaZ*F-R, *aac/aph*, *tcaR*, *IS256*	Penicillin G, Ampicillin
44	b	*qacJ*, *smr*	*BlaI*, *BlaZ*1-2, *vanA*, *IS256*	Penicillin G, Ampicillin
46	c	*qacAB*, *qacJ*, *smr*	*mecA*, *BlaR*, *BlaI*, *BlaZ*1-2, *BlaZ*F-R, *tcaR*	Penicillin G, Ampicillin, Levofloxacin, Ofloxacin, Norfloxacin
47	c		*Bla*I, *Bla*Z1-2, *Bla*ZF-R, *tcaR*	Penicillin G, Ampicillin, Cefoxitin
97	d		*BlaI*, *BlaZ*1-2, *ant*, *tcaR*, *IS256*	Cefoxitin, Fusidic Acid
100	d		*BlaI*, *BlaZ*1-2, *BlaZ*F-R, *tcaR*, *IS256*	Tetracycline
101	d	*qacAB*, *smr*	mec*A*, *BlaI*, *BlaZ*1-2, *BlaZ*F-R, *tcaR*	Penicillin G, Ampicillin, Teicoplanin
102	d		*mecA*, *BlaR*, *BlaI*, *BlaZ*1-2, *BlaZ*F-R, *tcaR*, *vanA*, *tetK*, *IS256*	Penicillin G, Ampicillin, Teicoplanin, Tetracycline, Erythromycin
112	e		*BlaI*, *BlaZ*1-2, *BlaZ*F-R, *tcaR*	Penicillin G, Ampicillin
114	e		*BlaI*, *BlaZ*1-2, *BlaZ*F-R, *tcaR*	Penicillin G, Ampicillin
123	e		*BlaI*, *BlaZ*1-2, *BlaZ*F-R, *tcaR*	Penicillin G, Ampicillin
124	e		*BlaI*, *BlaZ*1-2, *BlaZ*F-R, *tcaR*	Penicillin G, Ampicillin, Gentamicin, Tobramycin, Cefoxitin, Ofloxacin, Fusidic Acid
131	e		*IS257*	Teicoplanin, Cefoxitin
167	f		*tcaR*, *IS256*	Teicoplanin
168	f		*IS256*	Cefoxitin
190	f			Teicoplanin
194	f	*qacC*, *qacJ*, *smr*	*mecA*, *tcaR*, *vanA*, *IS256*	Penicillin G, Ampicillin, Tobramycin, Teicoplanin
218	g			Ampicillin
Total Strain Number (%)		6/28 (21.4%)	25/28 (89.2%)	27/28 (96.4%)

## Data Availability

The data presented in this study are available on request from the corresponding author.
